# Trefoil factor family 3 (TFF3) and migration inducting gene 7 (MIG7) as molecular markers for early detection of endometrial carcinoma micro metastases

**DOI:** 10.1186/s12885-026-15783-z

**Published:** 2026-03-26

**Authors:** Dina Aly Abdelaziz, Amal K. Seleem, Abdel Aziz A. El Refaeey, Lamiaa A. Barakat, Mohamed M. Tawfik

**Affiliations:** 1 Faculty of Science, Port-Said University, Port Said, Egypt; 2 Faculty of Medicine, Mansoura University, Mansoura, Egypt; 3 Sherbin, Egypt

**Keywords:** Endometrial carcinoma, Gene expression, TFF3 protein level, TFF3 gene expression, MIG7 gene expression

## Abstract

**Background:**

Early detection of micrometastatic disease in endometrial carcinoma (EC) remains a major clinical challenge. This study evaluated Migration Inducing Gene 7 (MIG7) and Trefoil Factor Family 3 (TFF3) as minimally invasive biomarkers for EC progression.

**Methods:**

MIG7 and TFF3 gene expression and circulating TFF3 protein levels were analyzed in 75 subjects: healthy controls (*n* = 25), non-metastatic EC (*n* = 25), and metastatic EC (*n* = 25). Diagnostic performance was assessed using ROC analysis and logistic regression.

**Results:**

A significant stepwise increase was observed from controls to metastatic EC in MIG7 expression (0.69 vs. 1.97), TFF3 expression (0.38 vs. 2.03), and TFF3 protein levels (0.41 vs. 6.09; *P* < 0.001). Diagnostic accuracy was high, with AUC values of 0.945, 0.998, and 0.999, respectively. TFF3 gene expression showed the highest balanced performance (96% sensitivity, 100% specificity). MIG7 and TFF3 expression were strongly correlated (*r* = 0.818, *P* < 0.001). All markers independently predicted metastatic disease (*P* < 0.05).

**Conclusion:**

MIG7 and TFF3 are robust minimally invasive biomarkers for EC detection and metastatic risk stratification.

**Supplementary Information:**

The online version contains supplementary material available at 10.1186/s12885-026-15783-z.

## Introduction

Uterine corpus cancer is the leading gynecologic malignancy among American women, with over 60,000 new cases and nearly 11,000 deaths anticipated annually. Most of these cancers are endometrial carcinomas, with less than 10% being sarcomas. Endometrioid carcinomas constitute over 83% of uterine corpus cancers, while the more aggressive serious and papillary serous carcinomas account for 4% to 6%, and clear cell carcinomas for 1% to 2% [1].

It is crucial to distinguish type 1 endometrioid from type 2 serous endometrial carcinomas and other aggressive non-endometrioid carcinoma histotypes for effective understanding, management, and prevention. The development of most endometrial endometrioid carcinomas begins with continuous endometrial proliferation, driven by estrogen without the counterbalance of progesterone, leading to various stages of endometrial hyperplasia (EH) [2]. Endometrial carcinoma is the most common type of uterine malignancy, with increasing incidence globally. While early-stage disease is often curable, the presence of micrometastases—small clusters of cancer cells that have spread beyond the primary tumor but are undetectable by conventional imaging or histopathology—poses a significant clinical challenge. These micrometastases can lead to disease recurrence and progression, even in patients initially diagnosed with early-stage cancer. Detecting micrometastases is crucial for accurate staging and tailoring treatment strategies. Their presence may necessitate more aggressive adjuvant therapy, such as chemotherapy or extended lymphadenectomy, which would not be considered based on standard staging alone. However, current diagnostic tools lack the sensitivity to reliably identify micrometastatic disease. Recent advances in molecular diagnostics are beginning to address this gap. Circulating tumor DNA (ctDNA) analysis is emerging as a promising tool for detecting minimal residual disease and micrometastases, offering a non-invasive method to monitor tumor dynamics and guide adjuvant therapy decisions [3]. molecular biomarkers capable of detecting micrometastatic spread through minimally invasive methods, such as blood-based assays, are urgently needed. This study investigates the potential of Trefoil Factor Family 3 (TFF3) and Migration Inducing Gene 7 (MIG7) as such biomarkers, crucial for screening, diagnosing, prognosing, and monitoring treatment of endometrial cancer micrometastasis, playing a key role in both primary and secondary prevention [4].

Trefoil family 3 (TFF3) is a small secretory peptide regulated by estrogen and known to be overexpressed in several hormone-responsive cancers, including breast and gastrointestinal malignancies. Its role in promoting epithelial restitution, cell migration, and resistance to apoptosis makes it a plausible candidate for involvement in tumor progression and micrometastasis. Prior studies have shown that TFF3 is significantly upregulated in endometrial carcinoma tissues and serum, suggesting its potential as a non-invasive biomarker. Recent research has further demonstrated that TFF3 contributes to oncogenic progression by enhancing cancer stem cell-like phenotypes, migration, and invasion in estrogen receptor-positive tumors Moreover, targeting TFF3 in combination with c-MET inhibitors has shown synergistic effects in reducing metastatic burden, underscoring its therapeutic potential [5].

TFF3, a small, secreted peptide regulated by estrogen, has been implicated in promoting EMT, a hallmark of metastasis, by downregulating E-cadherin, an adhesion molecule that maintains epithelial cell-cell contacts. This disruption in cellular adhesion enhances cell motility, a necessary feature for the metastatic spread of cancer cells [6]. Furthermore, TFF3 plays a significant role in angiogenesis and apoptosis resistance, two processes that facilitate tumor growth and survival in the metastatic niche [7]. By promoting these pathways, TFF3 enhances the tumor’s ability to grow and disseminate to distant organs. The regulation of TFF3 by estrogen is also of particular interest, as estrogen-driven signaling pathways are central to the pathophysiology of EC, which is often hormonally driven [8].

Migration-Inducing Gene 7 (MIG7) is a cysteine-rich protein originally identified in endometrial cancer cells treated with hepatocyte growth factor (HGF) and has since been implicated in promoting epithelial-mesenchymal transition (EMT), angiogenesis, and tumor invasiveness. Its expression is largely absent in normal tissues but elevated in various cancers, including ovarian, breast, and colon cancers. Importantly, MIG7 has been detected in circulating tumor cells, supporting its relevance in early metastatic spread. Recent studies have shown that MIG7 contributes to the formation of the pre-metastatic niche by modulating tumor-associated macrophages and enhancing vascular permeability, thereby facilitating micrometastasis [9].

MIG7 has been shown to contribute to tumor invasion and metastasis through the activation of the COX-2/PGE2 signaling pathway, which is well-established as a promoter of EMT and tumor cell motility [10]. MIG7 suppresses E-cadherin expression, further enhancing EMT and potentially contributing to vascular mimicry, a process whereby cancer cells adopt endothelial-like characteristics to promote blood vessel formation [11]. Additionally, the expression of MIG7 is induced by hepatocyte growth factor (HGF), a potent mitogen and motility factor that links MIG7 to the pathways that regulate cell migration, invasion, and angiogenesis [12]. These findings suggest that MIG7 is not only involved in the invasion-metastasis cascade but may also play a role in the creation of a pro-tumor microenvironment by facilitating angiogenesis and vascular mimicry. These findings underscore MIG7’s potential as both a biomarker and a therapeutic target in the early dissemination of endometrial carcinoma. Both TFF3 and MIG7 contribute to the molecular and cellular changes that underlie endometrial carcinoma progression. Their involvement in key pathways of EMT, angiogenesis, and apoptosis resistance highlights their dual role as both biomarkers and active participants in disease pathogenesis.

The aim of this study was to investigate the role of TFF3 as both a molecular marker and at the protein level for the early detection of endometrial carcinoma micrometastases. Additionally, the study aimed to examine the role of MIG7 as a molecular marker for the early detection of endometrial carcinoma micrometastases. The research sought to utilize blood samples for detection, offering a less invasive and more cost-effective alternative to biopsies or expensive imaging techniques.

## Materials and methods

### Study groups

This study involved 50 female patients and 25 control subjects, matched by age. The mean ages for the non-metastatic and metastatic groups were 49.0–73.0 and 48.0–83.0 years, respectively. Patients were classified according to the International Federation of Gynecology and Obstetrics (FIGO) staging systems into two groups: Non-metastatic endometrial carcinoma (stage I-II): 25 cases, Metastatic endometrial carcinoma (stage III-IV): 25 cases. All participants were randomly selected from the Oncology Center at Mansoura University Hospital, Egypt, between December 2019 and February 2022. This work was approved by the ethics and scientific committees of Port Said University and was in accordance with the ethical guidelines of the “Helsinki Declaration”.

Inclusion criteria for EC patients included: histologically confirmed diagnosis of endometrial carcinoma, age between 45 and 85 years, no prior chemotherapy or radiotherapy, and availability of complete clinical records. Exclusion criteria included: presence of other malignancies, autoimmune disorders, chronic inflammatory diseases, or refusal to provide informed consent.

For healthy controls, inclusion criteria were age-matched females with no history of malignancy, gynecological disorders, or chronic systemic illness. Exclusion criteria included: any abnormal findings on routine clinical examination, family history of endometrial or other cancers, or refusal to participate.

### Sample collection and laboratory tests

Five milliliters of peripheral venous blood were collected from patients in EDTA tubes, properly labeled, and transferred on ice to the Medical Biochemistry Department, where they were stored at -20 °C until processing. Another blood sample was collected in anticoagulant-free tubes, left at room temperature for 20 min, then centrifuged to separate serum, and stored at -20 °C until processing. Circulating Tumor Cells were separated by density-gradient centrifugation using Ficoll (Biocoll Separating Solution, Biochrom-Gmb, Cat no. L 6113, Germany) [13]. Total RNA was extracted from peripheral blood, with RNA concentration and purity assessed. cDNA was synthesized from RNA, followed by primer design and conditioning using PCR. Real-time PCR was used for quantification of TFF3 and MIG7. Serum samples were used to measure TFF3 levels by ELISA.

### Statistical analysis

The collected data was summarized, tabulated, and analyzed using SPSS software version 21 (IBM Corp. Released 2017. IBM SPSS Statistics for Windows, Version 25.0. Armonk, NY: IBM Corp.). Appropriate statistical tests were used to analyze the data, with a P value of less than 0.05 considered significant. The Area Under the Curve (AUC) values were calculated using the nonparametric method based on the trapezoidal rule. Prior to conducting multivariable logistic regression, multicollinearity among significant univariable predictors was assessed using the Variance Inflation Factor (VIF), and all VIF values were below 2.0, indicating no significant collinearity.

## Results

### Demographic and clinical characteristics of the study groups

The baseline demographic and clinical characteristics of the study groups are summarized in Table 1. No statistically significant differences were observed among control subjects, non-metastatic endometrial carcinoma (EC) patients, and metastatic EC patients with respect to age, menopausal status, parity, or number of pregnancies (*p* > 0.05 for all comparisons), indicating appropriate matching between groups. In contrast, body weight differed significantly across the study groups (*p* < 0.001), with higher values observed in both non-metastatic and metastatic EC patients compared with controls. This finding is consistent with the established association between increased body weight and EC and provides a relevant clinical context for subsequent molecular analyses.

**Table 1:** Baseline demographic and clinical characteristics of the study groups

Variable	Control (*n* = 25)	Non-metastatic EC (*n* = 25)	Metastatic EC (*n* = 25)	*P* value
Age (years), Mean ± SD	61.48 ± 7.98	61.92 ± 6.83	64.80 ± 7.67	> 0.05
Weight (kg), Mean ± SD	75.76 ± 9.20	93.24 ± 7.92	97.56 ± 6.12	< 0.001*
Menopausal status, n (%)				> 0.05
Premenopausal	4 (16.0)	2 (8.0)	0 (0.0)	
Postmenopausal	21 (84.0)	23 (92.0)	25 (100)	
Number of pregnancies Median (Min–Max)	3.0 (0.0–5.0)	3.0 (0.0–6.0)	4.0 (0.0–6.0)	> 0.05
Parous, n (%)	23 (92.0)	21 (84.0)	23 (92.0)	
Nulliparous, n (%)	2 (8.0)	4 (16.0)	2 (8.0)	

*EC* Endometrial carcinoma

^*^A *P* value < 0.05 was considered statistically significant

### Tumor characteristics and metastatic patterns in endometrial carcinoma patients

Tumor characteristics of EC patients are presented in Table 2. As expected, a highly significant difference in FIGO stage distribution was observed between the non-metastatic and metastatic groups (*p* < 0.001), with all non-metastatic cases classified as early-stage disease (FIGO I–II) and all metastatic cases presenting with advanced-stage disease (FIGO III–IV). Primary tumor size did not differ significantly between the two groups (*p* = 0.29), suggesting that metastatic spread in EC may not be solely dependent on tumor dimensions. Among metastatic cases, the lung, pelvic region, and peritoneum were the most frequently involved sites, while bone metastases were less common. This heterogeneous pattern reflects the variable dissemination behavior of advanced EC.

**Table 2:** Tumor characteristics and metastatic patterns in endometrial carcinoma patients

Variable	Category	Non-metastatic (Group IIA) *n* = 25	Metastatic (Group IIB) *n* = 25	*P* value
FIGO Stages	I–II	25 (100%)	0 (0%)	< 0.001*
	III–IV	0 (0%)	25 (100%)	
Tumor size (cm)	< 5 cm	16 (64%)	12 (48%)	
	≥ 5 cm	9 (36%)	13 (52%)	0.29
Metastatic sites	Lung	—	7 (28%)	
	Pelvic	—	7 (28%)	
	Peritoneum	—	7 (28%)	
	Bone	—	4 (16%)	

Data are presented as number (percentage)

^*^P value calculated using Fisher’s Exact test for Stages and Chi-square test for tumor size. Metastatic sites were analyzed descriptively in metastatic cases only

### Expression levels of MIG7 and TFF3 Genes and TFF3 protein

Significant differences in MIG7 gene expression, TFF3 gene expression, and TFF3 protein levels were observed across the control, non-metastatic EC, and metastatic EC groups (*p* < 0.001 for all markers; Table 3). Both MIG7 and TFF3 gene expression exhibited a clear stepwise increase from controls to non-metastatic EC and further to metastatic EC patients. A similar trend was observed for circulating TFF3 protein levels, which were markedly elevated in EC patients compared with healthy controls and reached the highest levels in metastatic disease. This consistent pattern indicates a strong association between increased expression of these markers and disease progression.

**Table 3:** MIG7 and TFF3 expression across study groups

Marker	Statistic	Control (Group I)	Non-metastatic EC (Group IIA)	Metastatic EC (Group IIB)	*P* value
MIG7 gene	Mean ± SD	0.69 ± 0.21	1.38 ± 0.39	1.97 ± 0.45	< 0.001
	Median (Min–Max)	0.7 (0.3–1.0)	1.5 (0.7–1.9)	1.9 (0.8–2.7)	
TFF3 gene	Mean ± SD	0.38 ± 0.16	1.28 ± 0.27	2.03 ± 0.28	< 0.001
	Median (Min–Max)	0.3 (0.2–0.9)	1.3 (0.6–1.8)	2.0 (1.5–2.8)	
TFF3 protein	Mean ± SD	0.41 ± 0.15	3.79 ± 3.05	6.09 ± 3.64	< 0.001
	Median (Min–Max)	0.4 (0.2–0.8)	2.5 (1.0–10.0)	7.6 (0.7–10.0)	

Values are expressed as mean ± SD and median (min–max). *P* < 0.05 was considered statistically significant

### Diagnostic performance of MIG7 and TFF3 in discriminating endometrial carcinoma from controls

**Fig. 1:**
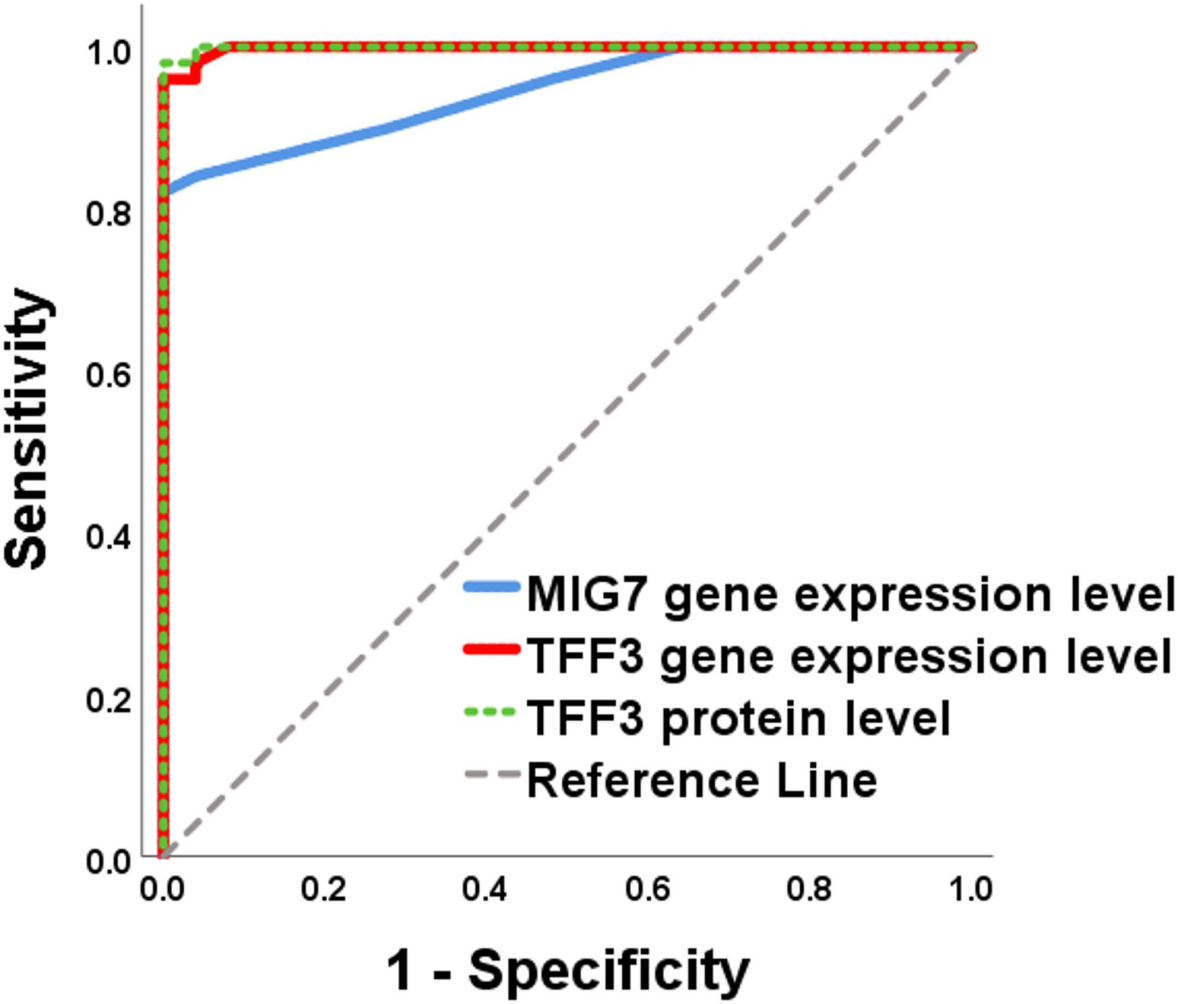
ROC Curve of MIG7, TFF3 gene expression and TFF3 protein level for discrimination between healthy subjects and patients with endometrial carcinoma

**Fig. 2:**
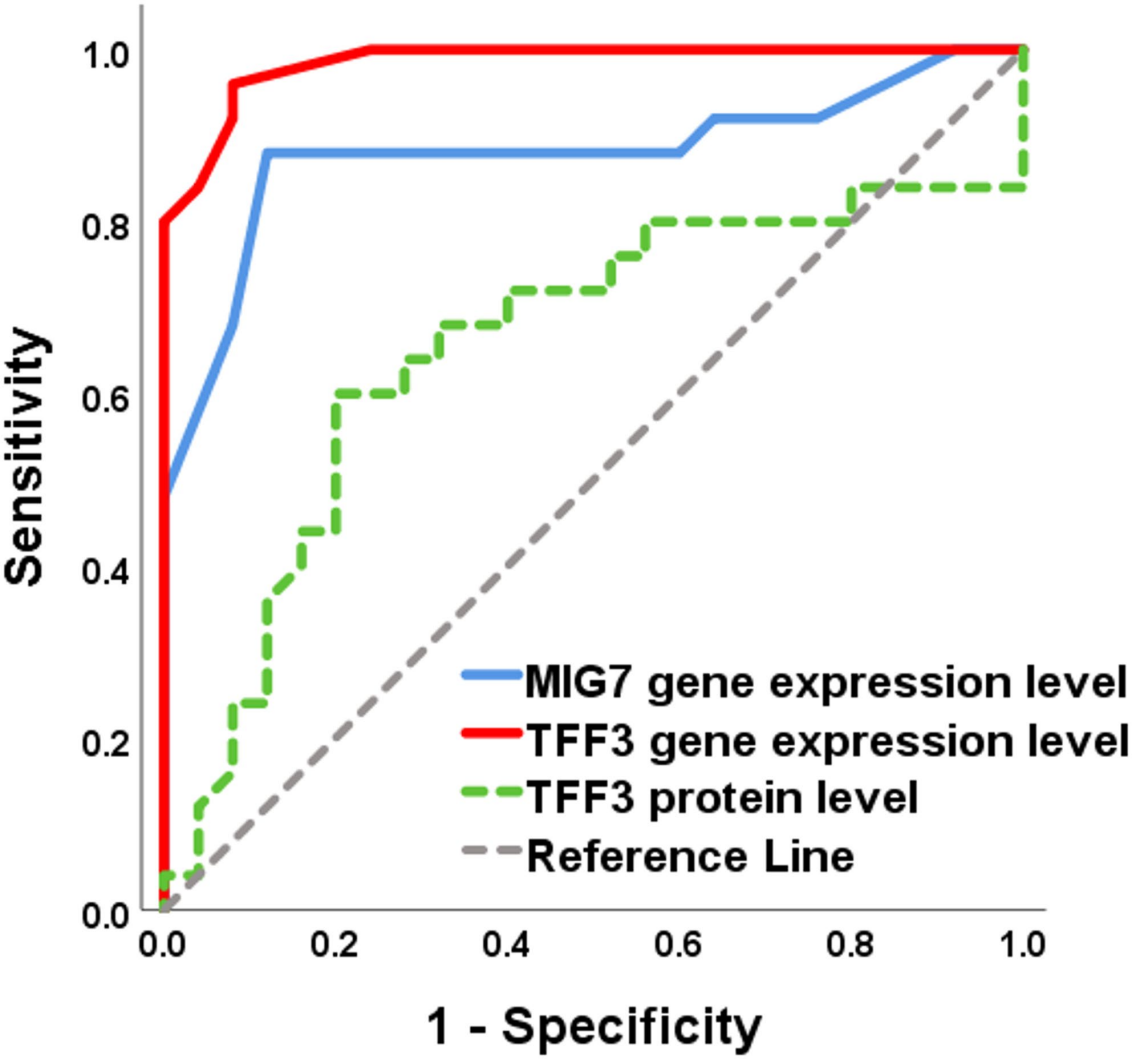
ROC Curve of MIG7, TFF3 gene expression and TFF3 protein level between non-metastatic and metastatic endometrial carcinoma

Receiver operating characteristic (ROC) curve analysis demonstrated excellent diagnostic performance of MIG7 gene expression, TFF3 gene expression, and TFF3 protein levels in distinguishing EC patients from healthy controls (Table 4; Fig. 1). All three biomarkers showed high accuracy, with statistically significant AUC values (*p* < 0.001). When discriminating metastatic from non-metastatic EC (Fig. 2), MIG7 and TFF3 gene expression retained strong diagnostic performance with significant AUC values. In contrast, TFF3 protein levels showed reduced discriminative ability in this comparison and did not reach statistical significance. These findings suggest that gene expression–based assays may be more informative than protein levels for identifying metastatic disease.

**Table 4:** Diagnostic performance of MIG7 and TFF3 for endometrial carcinoma and metastatic disease

Marker	Comparison	AUC (95% CI)	Cut-off	Sensitivity (%)	Specificity (%)	Accuracy (%)	*P* value
MIG7 gene	EC vs. Control	0.945 (0.898–0.991)	> 0.85	84	96	88.0	< 0.001
	Metastatic vs. Non-metastatic EC	0.880 (0.775–0.985)	> 1.75	88	88	88.0	< 0.001
TFF3 gene	EC vs. Control	0.998 (0.993–1.00)	> 0.65	96	100	97.3	< 0.001
	Metastatic vs. Non-metastatic EC	0.985 (0.961–1.00)	> 1.55	96	92	94.0	< 0.001
TFF3 protein	EC vs. Control	0.999 (0.997–1.00)	> 0.688	98	100	98.7	< 0.001
	Metastatic vs. Non-metastatic EC	0.654 (0.494–0.815)	> 2.74	72	60	66.0	0.061

ROC curve analysis was performed to assess diagnostic performance

*EC* Endometrial carcinoma

*P* values < 0.05 were considered statistically significant

### Correlation analysis of MIG7, TFF3 gene expression, and TFF3 protein levels

Correlation analysis revealed no significant associations between molecular marker expression and age or reproductive history (*p* > 0.05). In contrast, body weight showed significant positive correlations with MIG7 gene expression, TFF3 gene expression, and TFF3 protein levels (*p* < 0.001 for all). A strong positive correlation was observed between MIG7 and TFF3 gene expression, indicating coordinated regulation of these markers. In addition, TFF3 protein levels correlated significantly with both MIG7 and TFF3 gene expression, supporting the biological link between transcriptional activity and circulating protein levels (Table 5).

**Table 5:** Correlation between MIG7, TFF3 expression levels and clinical variables

Clinical variable	*MIG7 gene expression*		*TFF3 gene expression*		*TFF3 protein level*	
	*r*	*P* value	*r*	*P* value	*r*	*P* value
Age	0.150	0.199	0.162	0.166	0.014	0.907
Weight	0.603	< 0.001*	0.706	< 0.001*	0.500	< 0.001*
No. of pregnancies	0.123	0.294	0.175	0.134	0.124	0.289
TFF3 gene expression	0.818	< 0.001*	—	—	—	—
TFF3 protein level	0.534	< 0.001*	0.632	< 0.001*	—	—

Pearson’s correlation was used to assess associations between molecular markers and clinical variables

^*^P < 0.05 was considered statistically significant

### Univariable and multivariable logistic regression analysis

Univariable logistic regression analysis identified body weight, MIG7 gene expression, TFF3 gene expression, and TFF3 protein levels as significant predictors of metastatic disease. In the multivariable model, MIG7 gene expression, TFF3 gene expression, and TFF3 protein levels remained independently associated with metastasis, whereas body weight lost statistical significance after adjustment (Table 6).

**Table 6:** Univariable and multivariable logistic regression analysis of factors associated with metastatic disease

Variable	Univariable analysis			Multivariable analysis		
	*P* value	OR	95% CI	*P* value	OR	95% CI
Age	0.309	1.034	0.969–1.104	—	—	—
Weight	< 0.001*	1.325	1.163–1.510	0.092	1.012	0.946 − 1.018
Nulliparity	0.599	1.568	0.293–8.396	—	—	—
Menopausal status	0.093	4.571	0.776–26.92	—	—	—
MIG7 expression	0.002*	1.077	1.028–1.128	0.024*	1.088	1.012–1.252
TFF3 expression	0.003*	1.111	1.036–1.192	< 0.001*	1.358	1.172–1.575
TFF3 protein level	0.031*	1.232	1.019–1.489	0.019*	1.212	1.164–2.032

Univariable and multivariable logistic regression analyses were used to assess predictors of metastatic disease. ORs are presented with 95% CIs

^*^P < 0.05 was considered statistically significant

These results indicate that elevated expression of MIG7 and TFF3, at both gene and protein levels, represents an independent molecular signature associated with metastatic progression in endometrial carcinoma.

## Discussion

The present study provides comprehensive evidence supporting the potential diagnostic and prognostic utility of MIG7 and TFF3 at both gene and protein levels in endometrial carcinoma (EC). Our findings align with emerging data presented at ASCO 2023 and SGO 2024, which underscore the increasing relevance of molecular biomarkers in gynecologic malignancies for improved risk stratification and identification of aggressive tumor phenotypes. Although MIG7 and TFF3 are not yet incorporated into routine clinical practice, accumulating evidence suggests that these markers may contribute meaningfully to future biomarker panels aimed at early detection and metastatic risk assessment [14].

TFF3, a member of the trefoil factor family involved in epithelial restitution and mucosal protection, has been increasingly implicated in epithelial–mesenchymal transition (EMT), tumor invasion, and poor prognosis in endometrial and ovarian cancers. Similarly, MIG7 has been associated with tumor invasiveness and metastatic behavior, particularly in high-grade endometrial carcinoma [15]. In this context, the current study adds novel clinical evidence supporting the involvement of both markers in EC progression.

As shown in Table 1, demographic and clinical characteristics were largely comparable among study groups, with no significant differences in age, menopausal status, or reproductive history. Body weight, however, differed significantly, with higher values observed in EC patients, particularly those with metastatic disease. These findings are consistent with the well-established association between obesity and EC risk and progression and provide a relevant clinical background for interpreting molecular alterations observed in this cohort.

Tumor characteristics and metastatic patterns, summarized in Table 2, revealed a clear distinction between non-metastatic and metastatic EC cases, with all non-metastatic patients presenting at early FIGO stages (I–II) and all metastatic cases at advanced stages (III–IV). Tumor size did not differ significantly between groups, suggesting that metastatic spread may not be solely dependent on primary tumor dimensions. Metastatic dissemination most frequently involved the lung, pelvic region, and peritoneum, supporting previous reports that EC exhibits heterogeneous metastatic behavior with a predilection for intrapelvic and intraperitoneal spread [16]. These findings contrast with earlier observations by Lee et al. [17], emphasizing variability in disease presentation across populations.

At the molecular level, a stepwise and statistically significant increase in MIG7 gene expression, TFF3 gene expression, and TFF3 protein levels was observed from controls to non-metastatic EC and further to metastatic EC patients (Table 3). This progressive pattern strongly suggests a role for these markers in disease development and metastatic progression. MIG7 expression, which is negligible in normal tissues but markedly upregulated in malignant cells, has been implicated in cancer invasion through COX-2–PDE2 signaling and E-cadherin suppression, thereby promoting EMT. While MIG7 has been previously studied in epithelial ovarian cancer, this study represents, to our knowledge, the first clinical evaluation of MIG7 expression in endometrial carcinoma and its metastatic behavior [9, 18].

Similarly, elevated TFF3 gene and protein expression in EC patients is consistent with prior molecular and transcriptomic studies. Bignotti et al. demonstrated upregulation of TFF3 mRNA in endometrial carcinoma tissues, while Neubert et al. confirmed significantly higher TFF3 levels in EC compared with benign endometrial conditions, supporting its specificity as a malignant biomarker [19, 20]. Our findings extend these observations by demonstrating a clear association between TFF3 expression and metastatic disease status.

The diagnostic performance of MIG7 and TFF3 was further validated through ROC curve analysis (Table 4; Figs. 1 and 2). All three markers exhibited excellent accuracy in discriminating EC patients from healthy controls, with particularly high AUC values for TFF3 gene expression and protein levels. Importantly, when distinguishing metastatic from non-metastatic EC, MIG7 and TFF3 gene expression retained strong diagnostic performance, whereas TFF3 protein levels showed limited discriminatory ability. These findings highlight gene expression–based assays as more robust tools for metastatic risk assessment in EC.

Correlation analysis (Table 5) revealed significant positive associations between body weight and all molecular markers, reinforcing the link between obesity and molecular dysregulation in EC. Strong correlations between MIG7 and TFF3 gene expression, as well as between gene and protein expression levels, suggest coordinated regulatory mechanisms underlying tumor progression. In contrast, age and reproductive factors showed no significant associations, underscoring the specificity of these biomarkers to tumor biology rather than general demographic characteristics.

Multivariable logistic regression analysis (Table 6) further demonstrated that MIG7 gene expression, TFF3 gene expression, and TFF3 protein levels are independent predictors of metastatic disease, even after adjustment for potential confounders. These findings emphasize the clinical relevance of these markers as potential tools for identifying patients at higher risk of metastasis.

Beyond EC, elevated TFF3 expression has been reported in several malignancies, including gastric, colorectal, breast, thyroid, and hepatocellular carcinomas, where it correlates with tumor aggressiveness, lymph node involvement, and poor prognosis [21–26]. Collectively, these studies support the broader oncogenic relevance of TFF3 and reinforce its potential utility as a pan-cancer biomarker.

The strong association between increased body weight and elevated MIG7 and TFF3 expression observed in this study is further supported by epidemiological evidence. A meta-analysis by Jenabi and Poorolajal demonstrated significantly increased EC risk among overweight and obese individuals, highlighting obesity as a critical driver of disease development and progression [22]. These findings underscore the need for integrated clinical and molecular approaches to EC risk assessment.

Despite its strengths, this study has several limitations. The relatively small sample size and single-center design may limit generalizability. The cross-sectional nature of the study precludes assessment of causality or long-term prognostic value. Additionally, although TFF3 protein levels demonstrated excellent diagnostic accuracy for EC detection, their ability to discriminate metastatic status was less robust, warranting further investigation. Future multicenter and longitudinal studies incorporating molecular subtype stratification, as defined by TCGA classification systems, are needed to validate these findings and refine their clinical applicability [27, 28]. Moreover, evaluating combined biomarker panels may further enhance diagnostic accuracy and support personalized management strategies in endometrial carcinoma [29, 30].

## Conclusions

In this study, elevated levels of MIG7 gene expression, TFF3 gene expression, and TFF3 protein were observed in patients with endometrial carcinoma compared to healthy controls, with progressive increases from non‑metastatic to metastatic disease. These findings suggest that both MIG7 and TFF3 may serve as promising biomarkers for the early detection of micrometastases in endometrial carcinoma. The high sensitivity and specificity observed in this cohort highlight their potential diagnostic utility. However, these results should be interpreted with caution, as they represent preliminary evidence from a single‑center study. Further validation in larger, multicenter, and longitudinal cohorts is required to establish the clinical applicability of these biomarkers and to determine their role in guiding therapeutic decision‑making.

## Supplementary Information

Supplementary Material 1.

## Data Availability

The datasets used and/or analyzed during the current study are available from the corresponding author on reasonable request.

## Abbreviations

TFF3: Trefoil Factor Family 3
MIG7: Migration Inducing Gene 7
AUC: Area Under the Curve
RNA: Ribonucleic Acid
PCR: Polymerase Chain Reaction
ELISA: Enzyme-Linked Immunosorbent Assay
