# Supplementary material for: Trefoil factor family 3 (TFF3) and migration inducting gene 7 (MIG7) as molecular markers for early detection of endometrial carcinoma micro metastases
Source: BMC Cancer. 2026 Mar 26;26:457. doi: 10.1186/s12885-026-15783-z (PMC13064232; doi:10.1186/s12885-026-15783-z)
Supplement: Supplementary file 1 — Supplementary Material 1. [file 12885_2026_15783_MOESM1_ESM.pdf]

Metastatic endometrial Carcimoma

Control Groups

Patient ID	Age	No. of pregnancy	Stage	TFrg exp	MLgr... exp	TF3 ...	Primary Tumor Size	Metastatic Site	TAC	MDA	Weight
1	63	3	III	2.1	1.9	1.54	<5	Bone			
2	70	2	III	1.9	1.8	1.82	<5	Bone			95
3	83	4	III	1.7	2.1	1.92	>5	lung			100
4	48	6	IV	2.3	2.2	1.71	>5	Bone			102
5	59	5	III	2.1	2.0	1.73	<5	lung			99
6	69	0	IV	2	1.9	1.63	<5	pelvic			103
7	73	2	IV	1.9	1.8	1.12	<5	peritoneal			90
8	59	1	III	1.6	1.3	1.82	>5	lung			88
9	65	3	III	1.9	2.7	1.73	>5	peritoneal			105
10	73	4	IV	2.3	1.8	1.74	<5	pelvic			110
11	54	3	IV	2.2	1.9	1.01	<5	pelvic			107
12	75	4	III	2.1	2.5	1.71	<5	lung			99
13	62	5	IV	2.2	0.9	1.04	<5	lung			95
14	63	2	III	1.9	1.8	1.92	>5	pelvic			101
15	66	2	IV	2	2.3	1.83	>5	peritoneal			88
16	73	5	IV	1.7	1.4	1.74	>5	pelvic			91
17	57	6	IV	1.8	1.8	1.54	<5	lung			97
18	67	3	III	2	2.5	1.72	>5	pelvic			100
19	69	4	III	1.7	1.9	1.50	>5	peritoneal			101
20	58	4	IV	1.9	2.3	1.04	<5	~			102
21	70	0	III	2.3	2.4	1.03	>5	~			100
22	65	5	IV	2.4	2.2	9.98	>5	lung			97
23	60	4	III	1.9	1.9	7.97	<5	Bone			[illegible]
24	62	2	III	2.2	2.2	8.82	>5	pelvic			[illegible]
25	57	5	IV	2.8	2.3	1.34	>5	peritoneal			[illegible]

Control Groups

Patient ID	Age	No. of pregnancy	PERT	TFP	MI	FE mg/ml	OST	TAC	MDA	WT
	55	3	—	0.3	0.9	0.321	P.M			80
	63	5	—	0.4	0.4	0.234	M			81
	57	2	—	0.3	0.6	0.478	M			79
	70	—	—	0.7	0.7	0.556	M			75
	67	1	—	[illegible]	0.8	0.372	M			91
	59	3	—	0.4	0.6	0.281	M			77
	54	4	—	0.2	0.9	0.493	M			78
	75	2	—	0.4	0.3	0.472	M			75
	66	2	—	0.3	0.5	0.381	M			80
	49	3	—	0.2	0.7	0.253	P.M			69
	48	4	—	0.3	0.8	0.172	M			71
	62	2	—	0.4	0.7	0.382	M			73
	63	1	—	0.5	0.9	0.561	M			62
	75	3	—	0.5	0.9	0.472	M			80
	58	4	—	0.7	0.8	0.343	M			85
	67	5	—	0.2	0.7	0.291	M			87
	52	0	—	0.4	0.9	0.582	M			91
	70	2	—	0.3	1	0.771	M			83
	61	1	—	0.2	0.9	0.350	M			85
	73	3	—	0.3	0.8	0.230	M			90
	52	4	—	0.5	0.5	0.480	P.M			65
	67	2	—	0.3	0.8	0.875	M			70
	51	1	—	0.2	0.9	0.386	P.M			73
	60	3	—	0.4	0.3	0.471	M			74
	63	3	—	0.5	0.4	0.291	M			70

Non Metastatic endometrial Carcinoma ~~Control Groups~~

Patient ID	Age	No. of pregnancy	Stage	TFF exp	MI[illegible]	IGF (Elisa)	Primary tumour size	TAC	MDA	WT
1	55	3	I	0.9	1.3	0.9	<5	M		100
2	60	4	II	1	1.3	0.8	<5	M		98
3	57	2	II	1.2	[illegible]	0.7	<5	[illegible]		88
4	62	5	I	0.9	1.1	0.1	>5	[illegible]		87
5	67	1	I	1.3	1.2	1.71	<5	[illegible]		95
6	53	2	II	1.4	1.7	1.32	<5	[illegible]		97
7	73	6	I	1.5	1.5	1.25	<5	[illegible]		92
8	66	2	I	1.2	1.2	9.99	>5	M		100
9	63	—	II	1.3	1.5	2.81	>5	[illegible]		110
10	54	3	I	[illegible]	1.7	7.56	<5	[illegible]		83
11	70	4	I	[illegible]	1.8	6.81	<5	M		95
12	63	—	II	[illegible]	1.9	1.90	<5	M		97
13	59	3	I	1.3	1.5	9.88	<5	M		101
14	72	4	I	1.1	1.6	2.78	>5	M		75
15	69	2	II	1.3	1.4	[illegible]	>5	M		83
16	49	5	I	1.4	1.3	[illegible]	<5	M		89
17	56	—	I	1.3	0.9	8.74	<5	M		97
18	65	2	I	1.2	1.5	7.56	>5	M		81
19	62	1	II	1.5	1.6	8.81	>5	M		91
20	68	4	I	1.2	1.7	9.72	<5	M		92
21	64	6	II	1.3	1.6	1.10	<5	M		88
22	52	2	II	1.5	1.5	1.34	>5	[illegible]		101
23	73	—	I	1.5	1.7	1.25	<5	M		103
24	59	5	II	1.7	1.8	9.41	<5	M		98
25	57	3	I	1.4	1.9	8.32	>5	M		90
